# Editorial: Animal welfare assessment, Volume III

**DOI:** 10.3389/fvets.2023.1135932

**Published:** 2023-01-20

**Authors:** Edward Narayan

**Affiliations:** ^1^School of Agriculture and Food Sciences, Faculty of Science, The University of Queensland, St Lucia, QLD, Australia; ^2^Centre for Animal Science, Queensland Alliance for Agriculture and Food Innovation, The University of Queensland, St Lucia, QLD, Australia

**Keywords:** animal welfare, behavior, physiology, stress, assessment

The term “stress” is often regarded in the negative context, such as causing damaging effects on animal health and welfare. However, the underlying neuroendocrinological mechanisms of stress responses in animals are context-dependent and are mostly adaptive to change. The current fragile atmosphere of shifting perspectives in the animal production sector and societal awareness has placed increasing pressure on finding this balance between management practices that can reduce stress and, equally, improve farm animal productivity.

In this section edition, we focussed on discussing this balance between welfare and animal productivity. Getting the appropriate balance will be challenging, however it is possible. This requires a detailed understanding of the neuroendocrinological mechanisms of stress responsiveness in animals across crucial life-history stages and contexts. It also requires detailed studies applying novel physiological biomarkers to quantify the stress responses arising from the higher brain centers using techniques that can be readily adopted in the field.

We received 5 papers from animal welfare experts, veterinarians, animal physiologists and animal managers to generate a healthy discussion and showcase latest studies working toward finding the harmony between animal welfare and productivity. This is a volume III of the original special issue “Animal welfare assessment.”

In 2022 Edition of this Research Topic, we show a collection of 5 peer reviewed articles which highlight the different advancements in the fields of animal welfare and behavior.

The first manuscript by Ma et al. demonstrated a simple, objective, and reliable welfare assessment tool, coined the Animal Welfare Assessment Grid (AWAG) for application in South Korea Zoos. The AWAG has for components including physical, psychological, environmental and procedural, and they incorporate animal welfare factors such as behavior, housing, restraint etc. Animals are given a 6-point Likert scale score and averaged data is used as an AWAG score for each zoo. The study included participation by 16 zoos selected for holding large cohort of animals, and the AWAG data showed large differences between zoos especially showing inadequate welfare. The AWAG tool could be crucial for improving animal welfare standards in South Korea Zoos.

In the second research, Chang et al. conducted an analysis on the debatable Research Topic of Greyhound racing in Australia by reviewing 6 years of Greyhound management data available on public domain. The researchers wanted to determine if/how Greyhound mortality and morbidity events could be benched marked. Data across three states of New South Wales, Victoria and Queensland were analyzed. Results showed inconsistency in report availability in some state(s). The researchers raised alarm of this lack of consistency in data reporting which will be necessary for accurate evaluation of whether animal welfare standards are being met in Australian greyhound racing industry. The researchers also recommended the development of a publicly available whole-of-life tracking for individual racing greyhounds.

The third paper by Malkani et al. was based on the welfare of dogs which the researchers evaluated using the Animal Welfare Assessment Grid (AWAG). Veterinary professionals were consulted to refine and improve the AWAG. Subject matter experts rated the validity of the factors for assessing dog welfare. Results showed the potential for AWAG to differentiate between healthy and sick dogs, and healthy and healthy dogs post elective surgery.

The fourth and final papers were from the researchers Mayes et al. who tested the welfare implications for sheep during live export by simulating stocking density and trough space. Merino weathers were housed under high or low stock density and monitored for 18 days. Results showed that higher stocking density ewes spent less time lying and increased agonistic social interactions. Live weights showed minor reduction at the end of trial however physiological parameters (fecal glucocorticoid metabolites and immune cells) were unaffected. In their corrigendum Mayes et al. provided correction to the y-axis of their figure showing the proportion of animals lying with head down across days.

Overall, this Research Topic highlights some of the recent developments in the broad field of animal welfare assessment.

## Author contributions

EN conceptualized this special topic and edited the manuscripts.

